# *In vitro* hypoxia-conditioned colon cancer cell lines derived from HCT116 and HT29 exhibit altered apoptosis susceptibility and a more angiogenic profile *in vivo*

**DOI:** 10.1038/sj.bjc.6602864

**Published:** 2005-12-06

**Authors:** K Yao, J A Gietema, S Shida, M Selvakumaran, X Fonrose, N B Haas, J Testa, P J O'Dwyer

**Affiliations:** 1 University of Pennsylvania Cancer Center, Philadelphia, 51 N. 39st street, MAB Suite 103, PA 19104, USA; 2 Groningen University Hospital, Groningen The Netherlands; 3 Fox Chase Cancer Center, Philadelphia, PA 19111, USA

**Keywords:** hypoxia, angiogenesis, colon cancer, cisplatin, apoptosis

## Abstract

Hypoxia is an important selective force in the clonal evolution of tumours. Through HIF-1 and other transcription factors combined with tumour-specific genetic alterations, hypoxia is a dominant factor in the angiogenic phenotype. Cellular adaptation to hypoxia is an important requirement of tumour progression independent of angiogenesis. The adaptive changes, insofar as they alter hypoxia-induced apoptosis, are likely to determine responsiveness to antiangiogenic strategies. To investigate this adaptation of tumour cells to hypoxia, we recreated *in vitro* the *in vivo* situation of chronic intermittent exposure to low-oxygen levels. The colon carcinoma cell lines HT29 and HCT116 were subjected to 40 episodes of sublethal hypoxia (4 h) three times a week. The resulting two hypoxia-conditioned cell lines have been maintained in culture for more than 2 years. In both cell lines changes in doubling times occurred: in HT29 an increase, and in HCT116 a decrease. Cell survival in response to hypoxia and to DNA damage differed strikingly in the two cell lines. The HT29 hypoxia-conditioned cells were more resistant than the parental line to a 24 h hypoxic challenge, while those from HCT116 surprisingly were more sensitive. Sensitivity to cisplatin *in vitro* was also significantly different for the hypoxia-conditioned compared with the parental lines, suggesting a change in pathways leading to apoptosis following DNA damage signaling. The growth of both conditioned cell lines *in vivo* as xenografts in immunodeficient (SCID) mice was more rapid than their parental lines, and was accompanied in each by evidence of enhanced vascular proliferation as a consequence of the hypoxia-conditioning. Thus the changes in apoptotic susceptibility were independent of altered angiogenesis. The derivation of these lines provides a model for events within hypoxic regions of colon cancers, and for the acquisition of resistance and sensitivity characteristics that may have therapeutic implications for the use of antiangiogenesis drugs.

Hypoxia within solid tumours has been shown to be a determinant of response to radiation and chemotherapy (Vaupel *et al*, 1989). A consequence of the disordered vasculature that characterizes growing tumours, hypoxia may occur locally as a consequence of distance from capillary nutrient supply or regionally by virtue of elevated tumour hydrostatic pressure or arteriolar vasoinstability impeding blood flow. Most solid tumours, even those 1–3 mm in diameter, exhibit hypoxic fractions that may range from 10 to 30% (Moulder and Rockwell, 1984). In tumours amenable to quantitation of hypoxia using an O_2_ electrode, the proportion of hypoxic cells is an important determinant of outcome (Bush *et al*, 1978; Brizel *et al*, 1997). Hypoxia also selects for more aggressive and metastatic cancer phenotypes that are associated with poor prognosis (Hockel *et al*, 1996).

A major driver of the biology of hypoxic tumours is the transcription factor HIF-1*α* (Carmeliet *et al*, 1998), which is responsible not alone for altered gene expression, but also may contribute to cancer progression and the development of a phenotype more refractory to therapy (Harris, 2001). HIF-1*α* increases angiogenesis through its regulation of VEGF expression (Forsythe *et al*, 1996), an effect that may be hypoxia-independent in some tumours (Semenza, 2003).

Cells rendered acutely hypoxic *in vivo* and *in vitro* show resistance to alkylating agents and platinum compounds (Kennedy *et al*, 1980; Tannock and Guttman, 1981; Teicher *et al*, 1990; Brown and Koong, 1991; Sakata *et al*, 1991). The basis for this resistance has been partially elucidated in recent years. In addition to HIF-1*α*, hypoxic cells have been shown to express higher levels of enzymes that are responsible for detoxication of cytotoxic drugs (O'Dwyer *et al*, 1994). Exposure of colon cancer cells to hypoxia results in altered expression of genes regulated by the AP-1 and NF-*κ*B transcription factor families, with recognized functional consequences (Yao *et al*, 1994; Yao and O'Dwyer, 1995), and not unexpectedly, microarray analyses reveal the induction of multiple transcripts from various functional groups (Koong *et al*, 2000). The relationship of gene expression changes to resistance to apoptosis induced by DNA-damaging agents is relevant to the clinical treatment of cancer. Several groups have shown that hypoxia itself may induce apoptosis (Yao *et al*, 1995; Shimizu *et al*, 1996; Amellem *et al*, 1997; Kim *et al*, 1997), and these observations form the basis of therapeutic efforts directed to the tumour vasculature (Chaplin and Hill, 2002). Therefore, we wished to understand the basis for resistance to apoptosis in hypoxic tumour cells.

Numerous examples exist in cancer therapeutics of the induction of drug resistance in cell lines by repeated exposure to the agent to which resistance is being induced (Kartner *et al*, 1983; Buller *et al*, 1987; Godwin *et al*, 1992). Such studies have served to identify resistance mechanisms that subsequently have been targets for the development of reversal strategies. We have adopted a similar approach to the identification of genes mediating hypoxic cell resistance by deriving cell lines through their repeated exposure to short periods of hypoxia (4 h), a duration shorter than that required to induce apoptosis. The colon adenocarcinoma cell lines HT29 (which is characterized by mutated p53), and HCT116 (which is characterized by wild-type p53 and microsatellite instability) were subjected to thrice-weekly treatments, and cell lines isolated after 40 treatments. In this manuscript, we present the divergent responses of these cell lines to hypoxia and to DNA-damaging agents. The conditioned cell lines were concordant, however, in their upregulation of angiogenesis, which correlated more closely with their growth characteristics *in vivo*. These findings support a role for these cell lines in exploring the basis for hypoxic cell sensitivity and resistance.

## MATERIALS AND METHODS

### Establishment of hypoxia-conditioned cell lines

HT29 and HCT116 human colon cancer cells were seeded in glass milk bottles and cultured in MEM medium supplemented with 10% FBS. The cells were exposed to hypoxia (0.1% O_2_) for 4 h and were then incubated in a standard culture environment (5%CO_2_, 95% air), at 37°C for 48–72 h. Cells were treated thrice weekly. The hypoxia-conditioned cell lines were derived by repeated exposure to hypoxia in this manner of both HT29 and HCT116, resulting in the generation of cell lines from HP10 to HP40 (from HT29) and HCP10 to HCP40 (from HCT116) respectively, following 40 exposures to hypoxia. A similar procedure was followed for the derivation of hypoxia-conditioned cells from a p53^−/−^ derivative of HCT116 (kindly provided by B Vogelstein, Johns Hopkins University).

### Cell growth parameters and cell cycle analysis

The cell doubling times were obtained by direct measurement of cell numbers. Cells (1 × 10^4^/dish) were plated in triplicate and incubated at 37°C in 5% CO_2_, 95% air. The cells were harvested at 24, 48, 72 and 96 h after plating. Doubling times were calculated using the formula: *N*/*N*_0_=*e*^kt^ where *N* is the cell number for a cell line at a particular time (*t*) and *N*_0_ is the corresponding cell number at time 0. The constant *k* was calculated for each cell line between 24 and 96 h, the period of time in which the cell growth rate was linear. The doubling time was then determined using the above formula with *N*/*N*_0_=2. For cell cycle analysis, cells (3 × 10^6^) were harvested by trypsinization, washed with cold PBS, and resuspended in 0.5 ml PBS, to which was added 5 ml cold 70% ethanol, followed by storage at −20°C until analysis. Before being analysed, cells were washed with PBS, resuspended in 455 *μ*l PBS, followed by addition of 25 *μ*l propidium iodide staining solution (1 mg ml^−1^) and 20 *μ*l RNase (1 mg ml^−1^). The suspension was then incubated at least 30 min in the dark, and analysed by flow cytometry.

### Identification of morphological differences produced by hypoxic conditioning cell lines

The hypoxia-conditioned cell lines HP40, HCP40, and control HT29 and HCT116 cells were evaluated for apparent morphological differences by light microscopy.

### Cytogenetic analysis

Cytogenetic analysis was performed on HT29 and HCT116, and the hypoxia-conditioned cell lines HP40 and HCP40. Metaphase cells were arrested by exposing cell overnight to colcemid (0.03 *μ*g ml^−1^). Chromosome spreads were prepared and G-banded according to standard procedures. Chromosome counts were obtained from at least 20 metaphase cells, and karyotypes were prepared from 5 to 10 of these cells, whenever possible. Chromosome identification and karyotype designations were in accordance with accepted guidelines for cancer cytogenetics (Mitelman, 1995).

### *In vitro* cytotoxicity assay

Cytotoxicity was determined using the 3-(4,5-dimethylthiazol-2-yl)-2,5-diphenyltetrazolium bromide (MTT) assay (Mosmann, 1983). For the drug cytotoxicity assay, cells were plated in 150 *μ*l of medium in each well of 96-well plates. Following overnight incubation, cells were exposed to various concentrations of cisplatin or oxaliplatin. Following 72 h incubation, 40 *μ*l of 5 mg ml^−1^ MTT was added per well. After 2 h at 37°C, the cells were lysed by adding 100 *μ*l of 20% (w v^−1^) SDS, and 50% (v v^−1^) *N*,*N*-dimethylformamide (pH 4.7) and incubated 3 h at 37°C. The absorbance at 570 nm was determined using a microplate reader (Elx800, BioTek Instruments Inc.). The reported values are the result of duplicate determinations. For cytotoxicity under hypoxia, cells were plated at a density of 3000 cells well^−1^ in 96-well plates. The plated cells were incubated overnight at 37°C, in 5% CO_2_ and 95% air. The cells were exposed to cisplatin or oxaliplatin, or to no drug for the hypoxic survival experiments, and placed in the anaerobic chamber (Forma Scientific Inc., Waltham, MA, USA) which was gassed using oxygen-poor (less than 1 part per 10 billion) 95% N_2_ and 5% CO_2_. After a 24 h incubation in the anaerobic chamber, cells were transferred to the incubator at 37°C, 5% CO_2_ and 95% air for additional 48 h, after which the MTT assay was performed as described above. The data shown in MTT assay are means±s.d. of two independent experiments carried out in triplicate. The statistical significance of the difference between control and hypoxia-treated with or without drug group was assessed by the two-sample Student's *t* test between two paired samples.

### TUNEL assay

Quantitation of apoptotic cells under hypoxic and oxic conditions was obtained using the TUNEL assay, which was performed according to the protocol of the Cell Death Detection Kit (Roche, Piscataway, NJ, USA). Cells were plated in glass petri dishes at a density of 1 × 10^6^/dish. Following overnight incubation under oxic conditions, the cells were incubated in the anaerobic chamber for 24 h. They were harvested, washed in PBS/1% BSA at 4°C, and fixed using a freshly prepared paraformaldehyde solution (4% in PBS, pH 7.4). After washing with PBS, cells were then treated with permeabilisation solution (0.1% Triton X-100 in 1% sodium citrate) for 2 min on ice. The cells were washed with twice with PBS, labeled with the TUNEL reaction mixture, and incubated for 60 min at 37°C in a humidified atmosphere in the dark. The reaction was stopped by adding 500 *μ*l of PBS. The apoptotic cells were quantitated by flow cytometry.

### Cell invasion assay

The capacity of the cell lines to exhibit invasive characteristics was determined using a Matrigel Invasion Chamber following the manufacturer's procedure (BD Biosciences, Bedford, MA, USA). Briefly, 1.25 × 10^5^ cells ml^−1^ were seeded in each chamber of a six-well dish. The chambers were prehydrated for 2 h in an incubator at 37°C in 5% CO_2_ and 95% air. After adding the cells, the Matrigel invasion chambers were incubated for a further 22 h at 37°C in 5% CO_2_/95% air, after which cells were fixed and stained by Coomassie blue solution for 30 min, and washed three times in PBS. After air-drying, the cells were counted. Control chambers without Matrigel were used to estimate migration rates. The invasion index was calculated as the ratio of cell numbers invading/migrating, and the hypoxia-conditioned cells were compared to their parental cell lines.

### Growth of the Conditioned cell lines *in vivo*

Adult (8–10 weeks of age) female C.B.17 SCID mice were purchased from Charles River Laboratories (Wilmington, MA, USA) for *in vivo* studies. The colon carcinoma cell lines HT29 and HCT116 and their respective hypoxia-conditioned cell lines HP40 and HCP40 were grown as a monolayer, and then trypsinised, washed, and resuspended in PBS. tumours were generated by subcutaneous injection of 1 × 10^7^ cells in 100 *μ*l PBS into the left flank of SCID mice. In one experiment, the hypoxia resistant cell lines HP40 and HCP40 were cultured in hypoxic conditions for 6 h 1 day prior to implantation, while in the second, the cells were used without further treatment. Subsequent tumour size was measured every 3 days using Vernier calipers, and the tumour volume calculated using the formula 1/2 (length^2^ × width). For tumour weight measurement and histochemical staining, tumour-bearing mice were killed on day 33, and tumours removed and snap frozen. Each experimental group consisted of six to eight SCID mice. The data shown are means±s.d. of mice in each group. All the animal experiments were performed in compliance with the NIH Guide for the Care and Use of Laboratory Animals and approved by the Institutional Animal Care and Use Committee.

### Northern blotting

Total RNA was isolated using the Trizol reagent (Gibco BRL, Grand Island, NY, USA). Briefly, 10^7^ cells were lysed in 3 ml Trizol reagent and incubated for 5 min at room temperature. After adding 0.6 ml of chloroform, the tube was vortexed and incubated at room temperature for 5 min. The sample was centrifuged at 12 000 **g** for 15 min at 4°C. The aqueous phase was mixed with 1.5 ml of isopropyl alcohol to precipitate the RNA, and again centrifuged at 12 000 **g** for 15 min at 4°C. The RNA pellet was washed once with 70% ethanol, and dissolved in DEPC-treated water. RNA was separated by electrophoresis in a 1% agarose denaturing gel. The RNA was transferred to nylon membranes, and hybridized to a 550 bp cDNA VEGF probe, or a 780 bp cDNA HIF-1*α* probe. A *β*-actin probe was used a loading control. Membranes were washed for 1 h in 2 × SSC containing 0.5% SDS and at 60°C, followed by 1 h in 0.1 × SSC containing 0.5% SDS. The filters were exposed to X-ray film at 70°C for 1–5 days.

### Immunohistochemistry

The frozen-sections fixed in cold acetone. The slides were treated for antigen retrieval by boiling for 10 min with 0.01 M citrate buffer pH 6.0. After antigen retrieval, slides were blocked with 1% BSA/Tris-buffered saline for 15 min. After a PBS wash, slides were incubated with rat anti-mouse CD31 (PECAM-1) monoclonal antibody (1 : 200 dilution: BD Biosciences Pharmingen, San Diego, CA, USA) at 4°C overnight, followed by incubation with biotinylated anti-rat antibody (Dako Corporation, Carpinteria, CA, USA) and horseradish peroxidase-conjugated streptavidin (Dako). Signals were visualized with diaminobenzidine as the substrate. The tissues were counterstained with hematoxylin. Normal rat antibody was used as a negative control. The quantitation of blood vessels in cross-sections of the tumours was based on the procedure described previously (Stephan *et al*, 2004).

### Statistics

Comparisons of quantitative measures between the cell lines were performed using *t*-tests, and *P*⩽0.05 accepted as significant.

## RESULTS

### Growth characteristics and morphology

Cell lines were derived by repeated exposure of the colon cancer cell line HT29 (a p53 mutated line, with intact mismatch repair, and a chromosomal instability [CIN^+^] phenotype) and HCT116 (a p53 wild-type line, with a defect in DNA mismatch repair but no chromosomal instability [CIN^−^]) to 4 h periods of hypoxia thrice weekly, resulting in the generation of cell lines HP40 and HCP40, respectively, each reflecting 40 exposures to hypoxia. The conditioned cell line HP40 displayed an increase in doubling time from 25.9 h in the parental line to 33.6 h (*P*<0.05) (Table 1). The conditioned cell line HCP40, by contrast, assumed a more proliferative program, and displayed a gradual decrease in doubling times from 28.5 h in the parental line to 20.5 h in HCP40, which was also significant (*P*<0.05). These changes were maintained for over 2 years in continuous culture, indicating their long-term effects. Thus repeated exposure of the cells to hypoxia led to stable changes in the proliferative characteristics of these colon cancer cells, in a manner that differed between the two cell lines.

The change in doubling time was not reflected in a marked change in cell cycle distribution under oxic conditions when measured 24 h after plating (Table 1). When exposed to hypoxia, however, the conditioned cells responded differently from parental cells. When HT29 cells are exposed to hypoxia, there was an apparent delay in progression into G2/M with a small increase in the proportion of cells in S (14–18%). This was not observed when HP40 cells were exposed to hypoxia, and cell cycle distribution was unchanged. On the other hand, a modest G1 arrest (51–62%) observed in the parental line (HCT116) did not occur in the conditioned cells (HCP40).

### Cytogenetics

The cytogenetic analysis of HCP40 and HP40 was conducted on a minimum of 20 metaphases per cell line. The karyotypes of the hypoxia-conditioned cell lines are identical to that of the parental cell line HCT116 and HT29 (data not shown). Thus gross alterations of chromosomal structure do not underlie the observed phenotypes.

### Apoptotic response of conditioned cells to hypoxia

**Figure 1:**
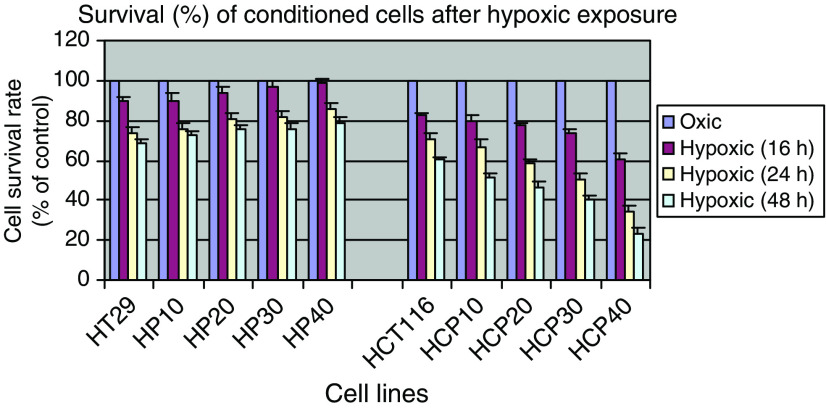
Survival of conditioned cells following various periods of hypoxia, as measured by the MTT assay. Cells were exposed to hypoxia for 4 h three times weekly, for up to 40 exposures (corresponding to HP10 to HP40 and HCP10 to HCP40), and cells harvested and stored after every 10 hypoxic treatments. For analysis of survival in hypoxia, cells were plated in 96-well plates, exposed to profound hypoxia for the durations indicated, and cultured under oxic conditions for an additional 72 h.

We and others have previously shown that hypoxia is a relatively strong inducer of apoptosis in cell lines *in vitro*, and have investigated the pathways involved (Pandey *et al*, 1999; Vasilevskaya and O'Dwyer, 1999; Garay *et al*, 2000). We studied the survival of the conditioned cells following varying durations of hypoxia and assessed outcome using the MTT assay (Figure 1). As with our previous studies of HT29 cells, an 8 h exposure to hypoxia had minimal effects on survival (data not shown). However, extending the hypoxic period to 16 or 24 h markedly compromised the survival of the cells. The HT29 series showed progressive acquired resistance to hypoxia from HP10 (the cell line derived after 10 exposures to hypoxia) to HP40, which demonstrated greater survival following a 24 h hypoxia exposure than HT29: 85.7±2.9 *vs* 73.8±1.7% (*P*<0.05). The opposite effect was observed with the HCT116-derived series: progressively greater susceptibility to hypoxia was observed to the point that the survival of HCP40 after 24 h of hypoxia (34.6±2.3%) was substantially less than that of the parental line (70.8±4.5%, *P*<0.05). Since the colorimetric assay may not distinguish between cell death and delayed growth, we performed a TUNEL assay to assess the proportion of cells in which apoptosis was induced. In this assay also (Table 2), the HT29-derived cells were more resistant: in HT29, 12.8% were apoptotic *vs* 2.1% in HP40 (*P*<0.05). By contrast, the HCP40 conditioned cells were substantially more susceptible: after an 8 h hypoxic exposure, 15.5% of the parental HCT116 cells were apoptotic *vs* 33.9% for HCP40 (*P*<0.05). Thus the HT29 derived cells had become more resistant to hypoxia, as expected, whereas the HCT116 series became more sensitive, despite a more aggressive phenotype in cell growth characteristics.

We hypothesized that the difference in p53 competence between the cell lines may account for the opposing directions of sensitivity to hypoxia upon repeated hypoxic stress. A similar procedure was followed to derive hypoxia-conditioned cells from HCT116/p53^−/−^, a p53-knockout cell line that has been well characterized in previous studies (Bunz *et al*, 1999). As anticipated, loss of p53 indeed influenced the direction of change in hypoxia sensitivity: the p53^−/−^ cells developed along the same lines as HT29, and became more resistant to apoptosis (Table 2).

### Sensitivity of conditioned cells to DNA-damaging agents

**Figure 2:**
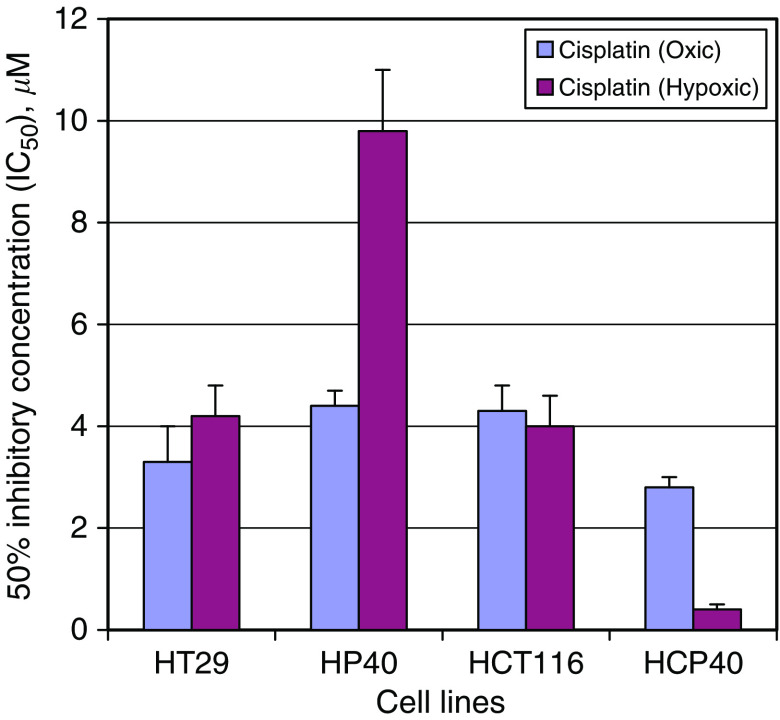
Sensitivity of hypoxia-conditioned cell lines to cisplatin as measured by the MTT assay. Cells were plated in 96-well plates, and after 18–24 h to allow adherence, a range of concentrations of cisplatin was added. For hypoxic incubation, the plates were immediately placed in the anaerobic chamber, left for 24 h at 37°C, and returned to an oxic environment for an additional 48 h. The MTT assay was performed at 72 h after initial exposure to the drugs in all cells. The one *asterisk* denotes the statistically significant difference between hypoxia-conditioned cell lines and its parental cell line with *P*-value <0.05.

To determine if the observed changes were specific to a hypoxic mechanism of cell death, or reflected a more general effect on apoptotic responses, we investigated the effects of a DNA-damaging agent. Cells were treated with varying concentrations of cisplatin under oxic and hypoxic conditions (Figure 2). Both parental cell lines are moderately sensitive to cisplatin when fully oxygenated. Hypoxia confers slight resistance to cisplatin in HT29 but not in HCT116 cells. Under oxic conditions, HT29-derived conditioned cells are slightly more resistant, and HCT116-derived cells more sensitive than their parental equivalents. A marked alteration in cisplatin sensitivity is observed in the conditioned cells under hypoxia, however. The HCP40 cells are 7.0-fold more sensitive to cisplatin (*P*<0.05), while HP40 are over 2.2-fold more resistant (*P*<0.05). The findings with oxaliplatin are very similar: HP40 is more resistant than HT29, while HCP40 is more sensitive than HCT116 (data not shown). Therefore, changes in sensitivity to platinum-induced and hypoxia-induced apoptosis vary among the cells in a concordant manner, implying that the change in apoptotic regulation in response to hypoxia conditioning occurs at a level downstream of the decision to commit to apoptosis.

### Invasive and migratory ability of the hypoxia-conditioned cells

The role of tumour cell invasion in determining the metastatic capacity of cancers has long been recognized (Liotta *et al*, 1982). Interactions with the cell matrix have been demonstrated to determine the behavior of *in vivo* models of human cancer, and efforts to target these interactions clinically have been made (Melchiori *et al*, 1992; Coussens *et al*, 2002; Egeblad and Werb, 2002). Recent data support a role for inhibition of specific matrix metalloproteinases (MMP's) in preventing invasion/metastasis (Holmbeck *et al*, 2003), and the potential to target relevant MMP's selectively may provide therapeutic targets. The invasive potential of hypoxia-conditioned cell lines was determined in an *in vitro* invasion chamber over a 22 h period (Table 3). The ability of the cells to invade Matrigel-coated filters is presented as the invasion index, the ratio of invasion through Matrigel divided by a control for cell motility. The results show that the HCP40 cells have a significantly higher invasion index compared to the parental HCT116 cells (7.1-fold increase, *P*<0.05), while HT29 and HP40 cells show no significant differences. These findings are concordant with observations on surface adhesion (greater invasive capacity and less adherence to surfaces).

### Growth of the conditioned cell lines *in vivo*

**Figure 3:**
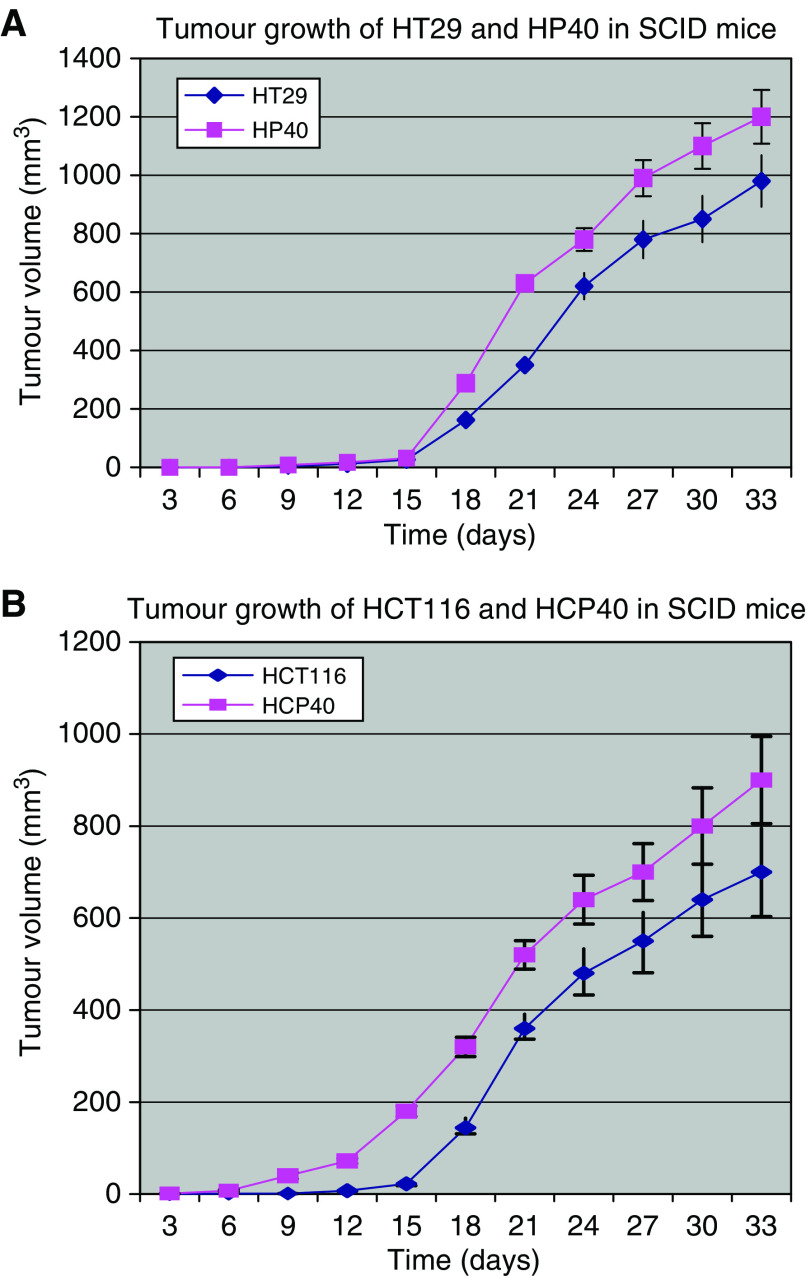
Tumour growth curve of HT29, HP40 (**A**), and HCT116, HCP40 (**B**) in SCID mice. tumours were generated by subcutaneous injection of 10^7^ cells in the left flank, and bidimensional measurements made every three days. The curves shown are the combined results of two separate experiments, the results of which were comparable.

We transferred the conditioned cell lines to an *in vivo* model by subcutaneous injection of SCID mice with 10^7^ cells in the flank, followed by tumour measurement twice weekly. The take rate was 100%. The growth characteristics of these tumours (six to eight animals/group) are shown in Figure 3. HCP40 grown *in vivo* recapitulates its *in vitro* growth characteristics growing more rapidly than the parental HCT116. The growth of HP40, however, is also more rapid than that of the parental HT29, despite its longer doubling time *in vitro*. Most interestingly, HCP40 starts its growth earliest of all the cell lines, suggesting that the conditioned line has acquired an ability to establish an optimal microenvironment, or conversely to evade environmental constraints upon such growth.

### Conditioned cell lines display a more angiogenic profile *in vivo*

**Figure 4:**
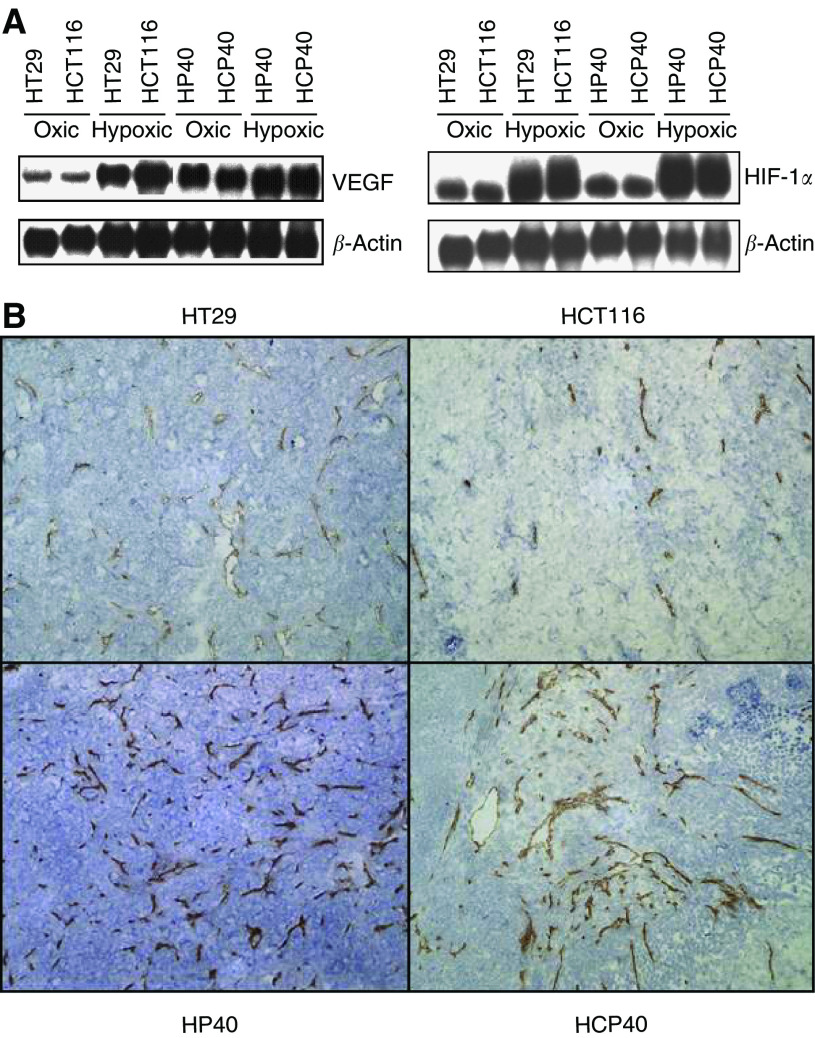
(**A**) Northern blot of RNA from the cell lines indicated, under oxic or hypoxic (16 h) conditions, probed with human VEGF and HIF-1*α* cDNAs. (**B**) Immunohistochemistry of formalin-fixed, paraffin-embedded tumour sections following 7 days' tumour growth of HT29, HP40, HCT116 and HCP40 implanted subcutaneously in scid mice. Sections of tumour were incubated with rat anti-mouse CD31, and developed by an immunoperoxidase technique.

We hypothesized, therefore, that the vascularization of the tumours *in vivo* may have been affected by the *in vitro* hypoxia conditioning. We analysed vascular endothelial growth factor (VEGF) expression in the tumour cells by northern analysis (Figure 4A). Induction of VEGF expression by hypoxia is observed in all cell lines, but both of the conditioned cell lines display upregulation of VEGF expression even under oxic conditions. A possible contribution of HIF-1*α* to this phenotype was explored in a preliminary fashion: in all cell lines HIF-1*α* is induced by hypoxia, but unlike VEGF, the baseline RNA expression of this gene is not elevated in oxic conditioned cells (Figure 4A). Western analysis showed similar profiles of HIF-1*α* protein expression (data not shown). This finding suggests that baseline elevation of VEGF transcription in these cells may be through VEGF-independent mechanisms. Sections of tumours grown *in vivo* were stained for microvessels using CD31 antibody (Figure 4B). The microvessel density was increased in tumours derived from both the conditioned cell lines derived from HT29 (13±1.8 *vs* 21±1.9 (mean±s.d.)) and from HCT116 (8±1.5 *vs* 17±2.1), and the increase is evident from the immunohistochemical image. The observation of increased vascularity in both models is consistent with their *in vivo* growth characteristics, in that both conditioned cell lines grow more rapidly than their parental lines (Figure 3). The increased susceptibility to apoptosis on the part of the HCT116-derived cell line does not therefore confer a growth disadvantage under nonstressed conditions (i.e. in the absence of a proapoptotic stimulus, such as would be provided by chemotherapy). This finding is consistent with the low levels of activity observed with single agent antiangiogenic treatment in most solid tumours.

## DISCUSSION

Angiogenesis in tumours represents the interaction of cancer cells, stromal cells and the endothelium of local blood vessels. The dysregulation of this interaction is reflected in the abnormal structure of the tumour vasculature, which results ultimately in areas of hypoxia. Hypoxic cells have altered responses to radiation and to cytotoxic drugs, and hypoxia has long been known to be a cause of resistance of cancer to treatment, be it with radiation or cytotoxic drugs (Bush *et al*, 1978; Kennedy *et al*, 1980; Tannock and Guttman, 1981; Vaupel *et al*, 1989; Teicher *et al*, 1990; Brown and Koong, 1991; Sakata *et al*, 1991; Brizel *et al*, 1997). Hypoxic cells also have the propensity to acquire genetic abnormalities that confer a ‘more malignant’ phenotype. For example, gene amplification was described to result from brief exposure of Chinese hamster ovary cells to hypoxia (Rice *et al*, 1986). Hypoxia can give rise to loss of p53 function in hypoxic transformed mouse embryonic fibroblasts, as a consequence of the acquisition of mutations (Graeber *et al*, 1996). More recently, some cell lines exposed to hypoxia are reported to be less susceptible to apoptosis (Dong and Wang, 2004; Zhang and Hill, 2004). Thus the hypoxic environment provides a vehicle for the clonal evolution of tumours to more advanced stages of carcinogenesis. The relationship between these observations and the resistant phenotype may usefully be explored using cell lines with acquired changes in responses to hypoxia. We report the characterization of a series of cell lines derived from two widely used colon carcinoma models, HT29 and HCT116. HT29 bears a mutation in one allele of p53, but based on p21 responses is functionally p53-deficient; HCT116 has normal p53 function, but is defective in mismatch repair resulting in the microsatellite instability phenotype (Rodrigues *et al*, 1990; Bhattacharyya *et al*, 1994).

Previous studies of acquired cytotoxic drug resistance have utilized cell lines with resistance acquired through repeated exposure to escalating concentrations of the drug of interest. This work has led to the isolation and characterization of numerous genes related to resistance, including the multiple drug resistance (mdr-1) gene and other members of the ATP-binding cassette membrane transporters, and participants in thiol-mediated drug detoxication (Ling *et al*, 1983; Hamilton *et al*, 1985; Wang and Tew, 1985). The protein products of these genes are now targeted by specific inhibitors in clinical trials. We have adopted a similar approach to the isolation of genes mediating hypoxic cell resistance. Colon cancer cells have been exposed repeatedly to sublethal periods of hypoxia, and cell lines isolated and characterized. We find that the resulting cell lines have acquired marked changes from the parental line, and that the conditioned cells are altered in their sensitivity both to hypoxia and to the cytotoxic drugs cisplatin and oxaliplatin. The concordant association of resistance and sensitivity to both of these stimuli is of some interest in that it is consistent with well-established data showing that hypoxic tumours are resistant to radiation also (Brown, 1999; Harris, 2001). It suggests that these stimuli may activate common response pathways to apoptosis, and further that the alteration in apoptosis sensitivity in the conditioned cell lines is downstream of the lesion that determines lethality, in the pathways mediating cellular responses to the damage. Apoptosis susceptibility is recognized to be critical to the effectiveness of anticancer therapy (Johnson *et al*, 1997; Johnstone *et al*, 2002). HIF-1*α* is a target for the development of drugs to reverse hypoxic resistance (Semenza, 2003): in this model it seems unlikely that HIF-1*α* is the major cause of the observed changes in susceptibility based on its profile of response to hypoxia, which does not differ as far as we can determine, between the sensitive and resistant lines. These cells may be a source therefore of novel targets to alter susceptibility to apoptosis in response to hypoxia and other stimuli.

Most striking in these results is the divergence of the fates of the cell lines derived by the same stimulus. Our initial purpose in developing these lines was to derive a hypoxia-resistant series of cell lines. The HT29-derived cell lines are indeed hypoxia-resistant, and capable of surviving prolonged extreme hypoxia. Clearly events that underlie this change are relevant to cells that fail to respond to antiangiogenic therapy. Unlike Dong and Wang (Dong and Wang, 2004), we did not find abnormalities in expression of bcl-2 family members to explain the resistance to apoptosis (data not shown). We were, however, able to show an effect of p53 in determining the development of resistance. HCT116 cells in which p53 was knocked out became resistant. Unexpectedly, the parental HCT116-derived lines proved more sensitive to the apoptosis-inducing effects of both hypoxia and cisplatin. Although the conditioning period was only 4 h, selection of a more sensitive population in the face of a toxic stimulus would be an unlikely violation of natural selection in the absence of a factor conferring a growth advantage. Presumably the accelerated growth rate provides this advantage, and raises the question of the link between growth rate and susceptibility to apoptosis. Such a link has been postulated through dysregulated E2F transcriptional activity increasing the content of both initiator and effector caspases (Nahle *et al*, 2002). A possible role for p53 is also suggested by the findings with HCT116 p53−/− cells, and data support a role for p53 in hypoxia-induced apoptosis (Leker *et al*, 2004). North *et al* (2005) have suggested that p53 may be involved in the angiogenic switch in some tumours. Altered growth characteristics in cell lines subjected to selection pressure has frequently been reported, but the underlying cause has rarely been identified, and generally the selected lines grow more slowly (Louie *et al*, 1992; Gibelli *et al*, 1996; Hochachka *et al*, 2002). We hypothesize that a mutational event is responsible for both increased susceptibility to apoptosis and the altered doubling time, consistent with the previous demonstration that the hypoxic environment facilitates mutational events (Graeber *et al*, 1996) and plausible especially in a cell line deficient in DNA mismatch repair. In this case, the development of a more apoptosis-prone cell line seems to be an evolutionary event in an environment to which tumour cells are commonly exposed *in vivo*. The means by which such intratumoural evolution is accelerated is under investigation.

Finally, this regimen of hypoxia conditioning *in vitro* altered the angiogenesis activity of the resulting tumours grown *in vivo*. Irrespective of susceptibility to apoptosis, both conditioned cell lines demonstrated an increase in VEGF expression, and when grown *in vivo*, enhanced angiogenesis, and this was associated with more rapid growth *in vivo* of tumours derived from each. VEGF expression is therefore dissociated from susceptibility to apoptosis. A contribution of VEGF to survival of cancer cells by antagonizing semaphorin signaling has recently been demonstrated to occur in lung and breast cancer cell lines (Castro-Rivera *et al*, 2004). These observations imply that a growth advantage of cells in a hypoxic environment is achieved in part by upregulation of proangiogenic factors, which in this model would appear to outweigh the importance of apoptosis susceptibility. These colon cancer cells provide therefore a model that may be relevant to the investigation of colon cancer therapy, especially in light of the recent demonstration of the effectiveness of antiangiogenic approaches in this disease.

## Figures and Tables

**Table 1:** Growth characteristics of HT29 and HCT116-derived cell lines before and after repeated 4 h exposures to hypoxia. Doubling times and cell cycle distribution in oxic conditions were measured under standard culture conditions

**Cell line**	**HT29**	**HP40**	**HCT 116**	**HCP40**
Doubling time (h)	25.9±2.3	33.8±1.9*	28.5 ±1.7	20.5±1.1*

*Cell cycle distribution*				
*Oxic conditions*				
G_0_/G_1_ (%)	72.5	71.5	50.6	54.8
G_2_/M (%)	8.5	9.5	23.5	20.2
S (%)	13.9	16.5	22.7	20.5

*Hypoxic conditions*				
G_0_/G_1_ (%)	59.3	74.1	62.4	47.5
G_2_/M (%)	10.7	11.5	10.2	9.4
S (%)	18.2	12.3	11.9	9.2

Cell cycle analysis under hypoxic conditions was conducted after a 24 h exposure to hypoxia, and included adherent and floating cells.

* denote a statistically significant difference between parental cell line and its hypoxia-conditioned derivative with *P-*value <0.05).

**Table 2:** Apoptosis (%) in hypoxia-conditioned cell lines

**Cell line**	**HCT116**	**HCP40**	**HT29**	**HP40**	**HCT116(−/−)**	**HCP40(−/−)**
Oxic	3.2±0.3	4.5±0.2	4.1±0.3	2.5±0.1	3.3±0.2	3.0±0.3
Hypoxia	15.5±1.1	33.9±2.7*	12.8±0.8	2.1±0.2*	7.5±0.7	3.9±0.5*

HT29 and HCT116 and their hypoxia-conditioned derivatives, HP40 and HCP40, were exposed to 24 h of hypoxia as described. In addition, apoptosis was measured in a matched pair of HCT116/p53^−/−^ cells conditioned and treated similarly.

* Denote a statistically significant difference between parental cell line and its hypoxia-conditioned derivative with *P-*value <0.05.

**Table 3:** Invasive and migratory ability of hypoxia-conditioned cells HP40 and HCP40 compared to their parental cells

**Cell line**	**HT29 (control cells)**	**HP40 (test cells)**
# cells invasion MIC (mean of triplicate)	4.3	5.3

% Invasion	4.6	6.4
Invasion Index	6.4/4.6=1.4	

**Cell line**	**HCT116 (control cells)**	**HCP40 (test cells)**
# cells invasion MIC (mean of triplicate)	8.0	56.0

% Invasion	3.9	28.0
Invasion Index	28.0/3.9=7.1*	

* denotes the statistically significant difference in invasion index between HP40 and HCP40.
